# Diagnostic Stewardship: A Systematic Review and Meta-analysis of Blood Collection Diversion Devices Used to Reduce Blood Culture Contamination and Improve the Accuracy of Diagnosis in Clinical Settings

**DOI:** 10.1093/ofid/ofad433

**Published:** 2023-08-11

**Authors:** Gustavo Yano Callado, Vivian Lin, Elizabeth Thottacherry, Tássia Aporta Marins, Marinês Dalla Valle Martino, Jorge L Salinas, Alexandre R Marra

**Affiliations:** Faculdade Israelita de Ciências da Saúde Albert Einstein, Hospital Israelita Albert Einstein, São Paulo, São Paulo, Brazil; Faculdade Israelita de Ciências da Saúde Albert Einstein, Hospital Israelita Albert Einstein, São Paulo, São Paulo, Brazil; Division of Infectious Diseases & Geographic Medicine, Stanford University, Stanford, California, USA; Faculdade de Medicina, Centro Universitário de Adamantina, Adamantina, São Paulo, Brazil; Faculdade Israelita de Ciências da Saúde Albert Einstein, Hospital Israelita Albert Einstein, São Paulo, São Paulo, Brazil; Division of Infectious Diseases & Geographic Medicine, Stanford University, Stanford, California, USA; Faculdade Israelita de Ciências da Saúde Albert Einstein, Hospital Israelita Albert Einstein, São Paulo, São Paulo, Brazil; Department of Internal Medicine, University of Iowa Carver College of Medicine, Iowa City, Iowa, USA

**Keywords:** antimicrobial stewardship, blood culture contamination, diagnostic stewardship, diversion techniques, initial specimen diversion device

## Abstract

**Background:**

Blood culture contamination may lead to misdiagnosis, overutilization of antibiotics, and prolonged length of stay. Blood specimen diversion devices can reduce contamination rates during blood culture collection procedures. We performed a systematic literature review and meta-analysis evaluating the influence of blood specimen diversion devices in blood culture contamination rates.

**Methods:**

We searched Medline, Cumulative Index to Nursing and Allied Health Literature, Embase, Cochrane, Scopus, and Web of Science, from database inception to 1 March 2023, for studies evaluating the impact of a diversion device on blood culture contamination. Blood culture contamination was a positive blood culture with microorganisms not representative of true bacteremia, but rather introduced during collection or processing the blood sample. Random-effects models were used to obtain pooled mean differences, and heterogeneity was assessed using the *I*^2^ test.

**Results:**

Of 1768 screened studies, 12 met inclusion criteria for this systematic literature review. Of them, 9 studies were included in the meta-analysis. Studies were substantially heterogeneous, but stratified analyses considering only high-quality studies revealed that venipuncture using a diversion device was associated with a significant reduction in blood culture contamination in comparison to the standard procedure of collection (pooled odds ratio [OR], 0.26 [95% confidence interval {CI}, .13–.54]; *I*^2^ = 19%). Furthermore, the stratified analysis showed that the adoption of a diversion device did not reduce the detection of true infection (pooled OR, 0.85 [95% CI, .65–1.11]; *I*^2^ = 0%).

**Conclusions:**

Blood culture diversion devices was associated with decreased contamination rates and could improve quality of care, reduce costs, and avoid unnecessary antibiotic use.

## Graphical Abstract

**Graphical Abstract:**
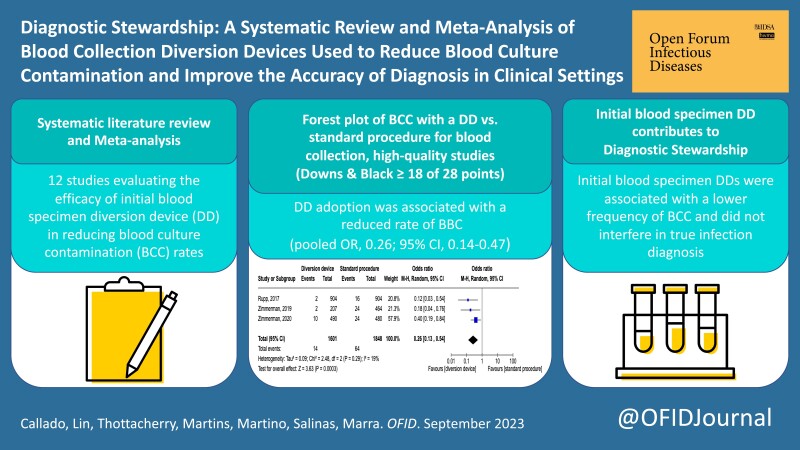

Around 30 million blood cultures are routinely collected annually in the United States for the diagnosis of bacteremia [1]. Most of these hospitals report a contamination rate of 2%–3% [1]. However, 20%–50% of all positive cultures are likely to be falsely positive [2, 3], leading to unnecessary broad-spectrum antibiotic therapy, prolonged hospital stay, and additional hospital costs [1, 4]. The American Society for Microbiology recommends a performance standard rate of <3% for blood culture contamination in medical institutions [5], but recent studies suggest that lower contamination rates may be achieved through the implementation of diagnostic stewardship elements [6]. In the last practical guideline for clinical microbiology laboratories, the Centers for Disease Control and Prevention registered its institutional recommendation of considering ≤1% as a new universal standard for allowable contamination rates, given that many facilities have already achieved this proportion following best practices [7, 8].

While standard procedures such as skin antisepsis and bottle disinfection have shown positive outcomes in reducing blood culture contamination rates [9], some studies show that further reduction may be achieved by discarding the initial blood draw portion, which potentially contains skin microorganisms [10–12]. In a recent review, 2 main procedures of diversion were presented: the open technique and the use of a diversion device [13, 14].

The implementation of a diversion device to remove potentially contaminated initial blood samples is being tested in some health institutions [13]. This idea converges with the concept of diagnostic stewardship, which aims to optimize diagnostic testing, thus reducing diagnostic error and consequent unneeded therapy [15]. This systematic literature review and meta-analysis aims to investigate the efficacy of initial blood specimen diversion devices in reducing blood culture contamination rates.

## METHODS

### Systematic Review and Search Strategies

This systematic literature review was conducted according to the Preferred Reporting Items for Systematic Reviews and Meta-Analysis (PRISMA) statement [16] and the Meta-analysis of Observational Studies in Epidemiology (MOOSE) guidelines [17]. This study was registered on the International Prospective Register of Systematic Reviews (PROSPERO; https://www.crd.york.ac.uk/PROSPERO/) on 20 March 2023 (CRD42023404440).

### Search Strategy

Our search strategy was developed in March 2023 with the assistance of a health sciences librarian with expertise in systematic reviews. We explored Medline (PubMed), Cumulative Index to Nursing and Allied Health Literature (CINAHL), Cochrane Central, Web of Science, and Scopus and Embase (Elsevier platform). The literature search included publications from database inception to 1 March 2023. The full PubMed search strategy (Supplementary Table 1) was adapted for the other databases. We searched only English-language literature.

Studies were included if they evaluated the impact of a blood diversion device on blood culture contamination. Blood culture contamination was defined as the presence of microorganisms in a blood culture that were not representative of a true bloodstream infection, but rather a false-positive result during collection or processing of the blood sample [4]. We excluded comments or reviews, studies without a control group, pilot studies, studies of devices not integrated into the needle or catheter, studies using an open diversion technique, or those solely performed in children.

**Figure 1.**
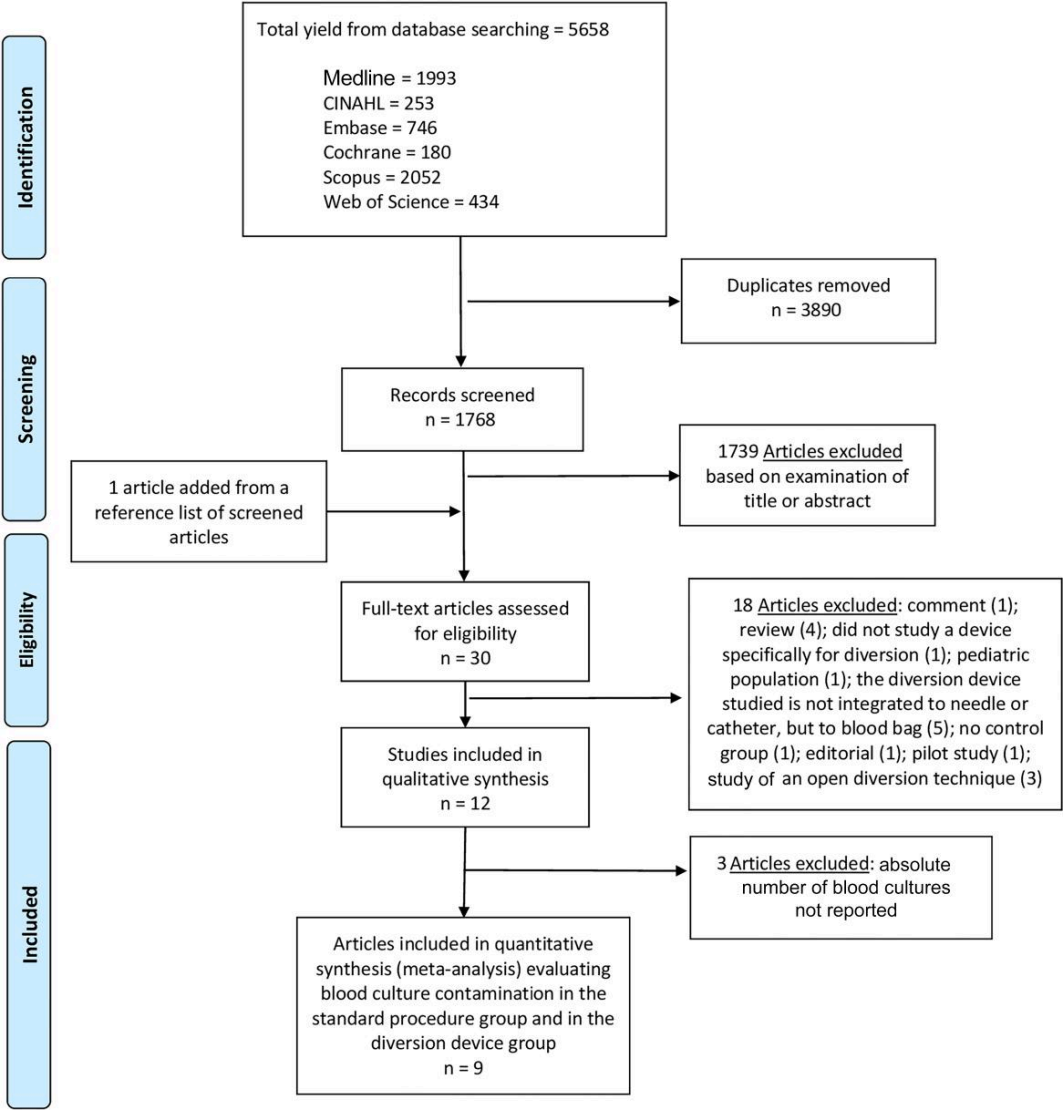
Literature search for articles that evaluated the impact of a blood diversion device in culture contamination. Abbreviation: Cumulative Index to Nursing and Allied Health Literature.

To filter the 1768 articles obtained from the databases, all titles and/or abstracts were examined (G. Y. C.), and those deemed unsuitable were excluded. After this first evaluation, all of the remaining articles were fully read, and 12 of them met the inclusion criteria and were included in the systematic review (Figure 1).

### Data Abstraction and Quality Assessment

Of 5 independent reviewers (G. Y. C., V. L., E. T., T. A. M., and A. R. M.), 2 abstracted data for each article using a standardized abstraction form (Supplementary Form 1). We recorded data regarding publication year, study period, design, population selection, setting, type of device, antisepsis procedure, blood culture contamination definition, outcomes, and financial analysis. We also collected information about the number of milliliters diverted, if they contained data on true bacteremia, and adjustment for potential confounders. Our primary outcome was the frequency of blood culture contamination in cultures collected with and without a diversion device.

We used the Downs and Black scale [18] to evaluate study quality. All questions of the original published scale were answered for each reviewed paper and the total score calculated. We adapted question 27 of the Downs and Black scale replacing the multiple-choices options with a yes/no answer. The maximum score was 28. The reviewers performed component quality analysis independently, and inconsistencies were resolved by discussion.

### Patient Consent Statement

The present investigation is a systematic literature review and meta-analysis of published data, so no patient informed consent was required.

### Statistical Analysis

To perform the meta-analysis with the extracted data, blood cultures collected with a standard procedure and through a diversion device were assessed using a random-effects model to estimate pooled odds ratios (ORs) and 95% confidence intervals (CIs) with weights as described by DerSimonian and Laird [19]. We performed stratified analyses considering study design, period of publication, duration, setting, tested device, detection system, collectors’ profession, number of participants in each group, antisepsis technique, study quality, and others (Supplementary Table 2). We excluded studies not registering the total number of blood cultures collected.

Heterogeneity between studies was evaluated using the *I*^2^ statistic and the Cochran Q statistic test. We used the Cochrane Review Manager (RevMan) Web edition 4.12.0. Publication bias was assessed using funnel plot with RevMan (Supplementary Figure 2) and the Egger test with Comprehensive Meta-Analysis version 4 software (Biostat Inc, Englewood, New Jersey).

## RESULTS

Of 30 articles reviewed in further detail, 12 studies met the inclusion criteria for this systematic literature review [20–31] (Figure 1). Of them, 5 were nonrandomized prospective controlled studies [20, 21, 27, 28, 30], 4 were quasi-experimental (pre-/postintervention comparison) studies [22, 23, 26, 29], 2 were randomized clinical trials [25, 31], and 1 was a case-control study [24] (Table 1). Ten were conducted in the United States [20–29] and 2 were conducted in Israel [30, 31]. All studies were performed between January 2013 and April 2021 [20–31]. Study duration varied from 3 to 24 months [20–31]. Of 12 studies, 8 evaluated Steripath (Magnolia Medical Technologies, Seattle, Washington) [21, 23–25, 27–30], 2 studies evaluated Kurin (Kurin, Inc, San Diego, California) [22, 26], 1 evaluated both Steripath and Kurin [20], and 1 used a biochemistry tube as a diversion device prior to blood inoculation [31]. The most common volume diverted by each device was 1.5–2 mL for Steripath [21, 23, 27–29] and 0.15 mL for Kurin [20, 22]. Regarding microbial detection techniques, 2 studies used BacTAlert (bioMérieux, Marcy-l’Étoile, France) and matrix-assisted laser desorption/ionization–time of flight (MALDI-TOF) [20, 27], 1 study used BacTAlert and polymerase chain reaction [25], 2 studies used BACTEC (Becton Dickinson, Franklin Lakes, New Jersey) [28, 29], 2 studies used BACTEC, MALDI-TOF, and standard biochemical methods [30, 31], 1 study used standard biochemical methods [26], and 4 studies did not report the adopted detection system [21–24].

**Table 1.** Summary of Study Characteristics

Study	Study Design	Setting	Diversion Device [mL Diverted]	Microbial Detection System	Samples Collected by	Patient Selection	Studied Groups	No. of Bcs	Contaminated Bcs	True Infection	Antisepsis Procedure	BC Contamination Definition	Adjusted Analysis for Potential Confouders	Studied Outcomes (LOS, Antibiotic Use, Mortality)	Financial Analysis	Downs and Black Score
Arenas, 2021, Texas, USA, Nov 2017–Feb 2019 [20]	Nonrandomized prospective controlled	Academic medical center	Two DDs: (A) One device (Magnolia Medical Technologies) [1–2 mL], preversion of Steripath; and (B) Kurin [0.15 mL]	BacTAlert 3D (bioMérieux) and Vitek MS MALDI-TOF (bioMérieux)	Nurses and nurse assistants	Conveniently in an ED	Two groups: SP and DDs (A for 5 mo and B for 12 mo)	4030; SP: 2054; device A: 664; device B: 1312	SP: 84 (4.1%), device A: 0 (0.0%), device B: 4 (0.3%); *P* value NR	NR	Skin: chlorhexidine Bottles: alcohol	Presence of 1 or more of the following organisms in only 1 bottle in a series of Bc sets: CoNS, *Micrococcus*, α-hemolytic viridans group streptococci, *Corynebacterium* sp, *Propionibacterium acnes*, and *Bacillus* sp	Linear regression (Poisson model)	NR	NR	13
Bell, 2018, Florida, USA, May–Nov 2016 [21]	Nonrandomized prospective controlled	4 community hospitals	Steripath (Magnolia Medical Technologies) [1.5–2 mL]	NR	Nurses and phlebotomists	Conveniently in 4 EDs	Two groups: SP and DD	41 685; SP: 35 392; DD: 6293	SP: 1246 (3.5%), DD: 38 (0.6%); *P* < .001	NR	Skin: scrub Bottles: chlorhexidine and alcohol	Presence of a skin-residing organism (not specified) in either the anaerobic or aerobic bottle but not both	NR	NR	During the study period, projected cost savings exceeded US$641 792 (approximately 184 prevented contaminations × US$3488 (US$8720 adjusted for charge-to-cost ratios of 40%)	14
Burnie, 2021, Ohio, USA, Jan 2018–Dec 2020 [22]	QE (pre-/postintervention comparison)	Community hospital	Kurin (Kurin, Inc) [0.15 mL]	NR	Nurses and technicians	Conveniently in an ED	Two groups: SP and DD	NR	SP: 2.9%, DD: 1.5%; *P* value NR	NR	Skin: chlorhexidine Bottles: alcohol	NR	NR	NR	Cost savings associated with the implementation of the DD ∼US$1.6 million in 2 y of implementation	9
Buzard, 2021, Kansas USA, Feb–Apr 2018 and Jun–Aug 2019 [23]	QE (pre-/postintervention comparison)	Community hospitals	Steripath [1.5–2 mL]	NR	Nurses	Conveniently in 3 EDs	Two groups: pre-DD and DD	3331; pre-DD: 1713; DD: 1618	Pre-DD: 128 (7.5%); DD: 42 (2.6%); *P* < .001	NR	Skin: alcohol Bottles: NR	Presence of 1 or more: CoNS, *Corynebacterium* spp, *Bacillus* spp other than *Bacillus anthracis*, *Propionibacterium acnes*, *Micrococcus* spp, viridans group streptococci, and *Clostridium perfringens*	NR	LOS (*P* = .7) and antibiotic duration (*P* = .19) not statistically different in both groups	Total hospital cost was US$1 120 000 in the pre-DD group vs US$383 690 in the DD group (difference of US$736 310)	15
Geisler, 2019, Washington, USA, Jan–Dec 2013 [24]	Case-control and survey	Academic medical center	Steripath [not specified]	NR	Phlebotomists	NR	135 patients with false-positive Bc vs 135 patients with true-negative Bc	NR	NR	NR	NR	NR	Kaplan-Meier estimates, survival curves, and a log-rank test	LOS: mean difference between false-positive Bc and true-negative Bc was 2.35 d (*P* = .008)	Each false-positive Bc resulted in incremental costs totaling US$6463, of which US$4818 was spent during hospitalization. DD would save US$186 per culture from the hospital perspective	19
Nielsen, 2022, Texas, USA, Oct 2015–Mar 2016 [25]	RCT	Military hospital	Steripath [not specified]	BacT/Alert (bioMérieux) and PCR (Verigene, Luminex)	Nurses, phlebotomists, and training service members	Conveniently in an ED	Two groups: SP and DD	1816; SP: 800; DD: 1016	SP: 53 (6.6%), DD: 7 (0.7%); *P* < .001	NR	Skin: chlorhexidine and alcohol Bottles: alcohol	Presence of CoNS, *Micrococcus* spp, certain *Corynebacterium* spp, *Actinomyces* spp, *Bacillus* spp, and *Cutibacterium acnes*	A 1-way analysis of variance comparison and Tukey HSD for unequal sample sizes with normal distribution	Antibiotic use: 31.4% reduction after DD and nucleic acid amplification test implementation	NR	15
O’Sullivan, 2019, Connecticut, USA, Apr–Jun 2017 [26]	QE (pre-/postintervention comparison)	Community hospital	Kurin Lock (Kurin) [not specified]	Standard biochemical methods	Phlebotomists	Conveniently	2 groups: SP and DD	NR	SP: 1.7%, DD: 0.4%; *P* value NR	NR	NR	NR	NR	NR	Projected cost savings of more than US$900 000/y or more than US$750 000/y adjusting for device costs	11
Povroznik, 2022, West Virginia, USA, Sep 2020–Apr 2021 [27]	Nonrandomized, prospective controlled	Community hospital	Steripath [1.5–2 mL]	BacTAlert 3D (bioMérieux) and Vitek 2 (bioMérieux)	ED staff, phlebotomists, and nurses	Conveniently in an ED, phlebotomy lab and acute-critical floors	2 groups: SP and DD	Hospital-wide: 5642; SP: 1011; DD: 4631. (ED: 3380; SP: 533; DD: 2847)	Hospital-wide SP: 41 (4.1%), DD: 36 (0.8%); *P* < .001 (ED: SP: 21 [3.9%], DD: 30 [1.1%]; *P* < .001)	NR	Skin: chlorhexidine Bottles: alcohol	Presence of a low-virulence commensal (CoNS, *Bacillus* spp, *Corynebacterium* spp, *Cutibacterium* spp, *Micrococcus* spp, or viridans group streptococci) in either the anaerobic or aerobic bottle but not both	NR	NR	DD had the potential to be cost-saving due to the small device cost relative to the mean US$2100 cost United Hospital Center attributes to contamination events	15
Rupp, 2017, Nebraska, USA, Nov 2015–Oct 2016 [28]	Nonrandomized prospective controlled	Academic medical center	Steripath [1.5–2 mL]	BACTEC 9240 system (Becton Dickinson), film array (Biofire) and automated identification and susceptibility testing (Microscan)	Phlebotomists	Conveniently in an ED	2 groups: SP and DD	1808; SP: 904; DD: 904	SP: 16 (1.8%), DD: 2 (0.2%); *P* = .001	SP: 69 (7.6%), DD: 65 (7.2%); *P* = .41	Skin: chlorhexidine and alcohol Bottles: alcohol	Presence of 1 or more of the following skin-residing organisms: CoNS, *Propionibacterium* spp, *Micrococcus* spp, viridians group streptococci, *Corynebacterium* spp, or *Bacillus* spp	Post hoc analysis and Poisson regression	NR	Estimation that the adoption of DD would lead to a cost avoidance of US$1.8 million/y at their institution (US$4850 per contaminated Bc), without considering the added cost of the device	22
Tompkins, 2022, California, USA, Mar 2019–Jan 2020 [29]	QE (pre-/postintervention comparison)	Academic medical center	Steripath [1.5–2 mL]	BD BACTEC Plus	Phlebotomists (SP and DD) and nurses (SP)	Conveniently in intensive care unit, ED and among inpatients	2 groups: SP and DD	23 372; SP: 12 170; DD: 11 202	SP: 171 (1.4%), DD: 1 (0.0%); *P* value NR	NR	NR	Detection in 1 of 4 bottles for matched sets or 1 of 2 bottles in both subsets for CoNS, viridans streptococci, *Corynebacterium* spp, *Cutibacterium acnes*, *Bacillus* spp, or *Micrococcus* spp	NR	NR	NR	17
Zimmerman, 2019, Jerusalem, Israel, Mar–Aug 2017 [30]	Nonrandomized prospective controlled	Academic medical center	Steripath [1–2 mL]	BD BACTEC Plus, MALDI-TOF (VITEK MS, bioMérieux) and standard biochemical methods	Phlebotomists and resident physicians	Conveniently in a department of medicine	2 groups: SP and DD	671; SP: 464; DD: 207	SP: 24 (5.2%), DD: 2 (1.0%); *P* = .008	SP: 44 (9.5%), DD: 16 (7.7%); *P* = .558	Skin: alcohol Bottles: alcohol	Growth of CoNS, corynebacteria, micrococci, or α-hemolytic streptococci	NR	NR	NR	18
Zimmerman, 2020, Jerusalem, Israel, Sep 2018–Feb 2019 [31]	RCT	Academic medical center	Sterile heparin lithium tube [not specified]	BD BACTEC Plus, MALDI-TOF, and standard biochemical methods	Nurses, phlebotomists, and resident physicians	Conveniently in an ED	2 groups: SP and DD	970; SP: 480; DD: 490	SP: 24 (5.0%), DD: 10 (2.0%); *P* = .01	SP: 26 (5.4%), DD: 18 (3.7%); *P* = .22	NR	Growth of CoNS, corynebacteria, micrococci, or α-hemolytic streptococci	A post hoc analysis neutralized demographic differences	LOS: mean reduction of 24 h in the DD group (*P* = .02) Mortality: lower mortality in the DD group (*P* = .03)	NR	23

Abbreviations: Bc, blood culture; CoNS, coagulase-negative staphylococci; DD, diversion device; ED, emergency department; HSD, honest significant difference; LOS, length of stay; MALDI-TOF, matrix-assisted laser desorption/ionization–time of flight; NR, not reported; PCR, polymerase chain reaction; RCT, randomized clinical trial; QE, quasi-experimental; SP, standard procedure; US$, United States dollars; USA, United States of America.

Sample collection was performed by nurses in 3 studies [20, 22, 23], phlebotomists in 3 studies [24, 26, 28], and both nurses and phlebotomists in 6 studies [21, 25, 27, 29–31]. Resident physicians also aided sample collection in 2 studies [30, 31].

All studies, except 1 that did not discuss patient selection [24], conveniently recruited participants to each intervention, with 7 studies [20–23, 25, 28, 31] recruiting from the emergency department. Two studies evaluated patients in the emergency department and in the intensive care unit [27, 29], 1 study evaluated a department of medicine [30], and 2 studies did not report the hospital units evaluated [24, 26]. Academic medical centers and community hospitals were the most referred settings, in 6 [20, 24, 28–31] and 5 studies [21–23, 26, 27], respectively. One of the studies was performed in a military hospital [25]. Two studies were conducted in >1 center [21, 23].

Most studies (11) reported the total number or the proportion of contaminated blood cultures [20–23, 25–31]. Three studies also shared the number of true infections [28, 30, 31]. Regarding antisepsis procedure information, 7 studies registered skin and bottle treatments before venipuncture [20–22, 25, 27, 28, 30], 1 study detailed only skin antisepsis [23], and 4 studies did not register the antisepsis procedure [24, 26, 29, 31]. Of the 8 studies that reported skin antisepsis [20–23, 25, 27, 28, 30], chlorhexidine and alcohol were the most used products, in 3 [20, 22, 27] and 2 studies [23, 30], respectively. Two other studies reported using both alcohol and chlorhexidine [25, 28] and 1 study used a “scrub” without specifying the component [21]. Of the 7 studies that reported the blood culture bottle top's disinfectant [20–22, 25, 27, 28, 30], 6 studies used only alcohol [20, 22, 25, 27, 28, 30], while another used both alcohol and chlorhexidine [21].

The definition of blood culture contamination was discussed in 9 studies [20, 21, 23, 25, 27–31], and was not mentioned in the remaining 3 [22, 24, 26]. Of the 9 studies, 1 did not specify the organisms considered as contaminants, only stating that the presence of a “skin-residing organism” in either the anaerobic or aerobic bottle (but not both) would define a blood culture as contaminated [21]. The other 8 studies listed the considered organisms. The most common microorganisms in the blood culture contamination definition were coagulase-negative staphylococci (CoNS) [20, 23, 25, 27–31], *Micrococcus* spp [20, 23, 25, 27–31], *Corynebacterium* spp [20, 23, 25, 27–31], α-hemolytic viridans group streptococci [20, 23, 27–31], and *Bacillus* spp [20, 23, 25, 27–29]. Less cited microorganisms include *Propionibacterium* spp [20, 23, 28], *Cutibacterium* spp [25, 27, 29], *Clostridium perfringens* [23], and *Actinomyces* spp [25].

Of the 9 studies that provided a definition of blood culture contamination, only 4 explicitly mentioned the required number of bottles for classifying contamination [20, 21, 27, 29]. Among these, 3 studies considered the presence of microorganisms in either an anaerobic or aerobic bottle, but not both, as an indication of blood culture contamination [20, 21, 27, 29]. The other study considered contamination when microorganisms were detected in 1 of 4 bottles for matched sets or 1 of 2 bottles in both subsets [20, 21, 27, 29].

Only one-quarter of the studies reported antibiotic use/duration, hospital length of stay (LOS), or mortality [23–25, 31]. The most reported outcome was LOS, in 3 studies [23, 24, 31]. One study reported no difference in LOS between diversion device and standard procedures (*P* = .7) [23]. Another 2 studies found significant reduction in LOS in the diversion device group (24 hours, *P* = .02) [31] and in the true-negative blood culture group (56 hours, *P* = .008) [24]. Two studies evaluated diversion device impact on antibiotic use [23, 25]. One of them did not find a statistically different antibiotic duration (*P* = .19) [23], whereas the other found a 31.4% reduction in antibiotic use after diversion device implementation [25]. The study reporting mortality data found a lower mortality in the diversion device group (*P* = .03) [31].

A financial analysis was performed by 7 of the 12 included studies [21–24, 26–28]. All of them reported a financial benefit of using diversion devices. Only 5 studies reported making an adjusted analysis for potential confounders [20, 24, 25, 28, 31]. The performed tests included post hoc analysis (2 studies) [28, 31], Poisson model (1 study) [20], Kaplan-Meier (1 study) [24], and 1-way analysis of variance with Tukey honest significant difference in another study [25].

Regarding quality assessment scores, 4 studies were considered of high quality (≥18 of the 28 possible points) per the Downs and Black quality tool [24, 28, 30, 31] (Supplementary Table 3). Five were considered fair (14–17 points) [21, 23, 25, 27, 29], and 3 were considered of poor quality (≤13 points) [20, 22, 26].

Overall, 9 studies, encompassing 83 325 collected blood cultures, evaluated contamination between those who used a diversion device and those who followed standard procedure of venipuncture (Supplementary Table 1 and Supplementary Figure 1) and were included in the meta-analysis [20, 21, 23, 25, 27–31]. Three studies that were included in the systematic review were not included in the meta-analysis because they did not report the total number of blood cultures [22, 24, 26]. Upon analysis of the 9 studies, the degree of heterogeneity was high (*P* < .001; *I*^2^ = 81%) (Supplementary Figure 1). However, even when considering 95% CIs, none of these studies demonstrated an advantage of using the standard procedure. To mitigate this high level of heterogeneity, we performed a stratified analysis that took into account studies that were based on supplementary parameters (Supplementary Table 2 and Supplementary Figure 1).

**Figure 2.**
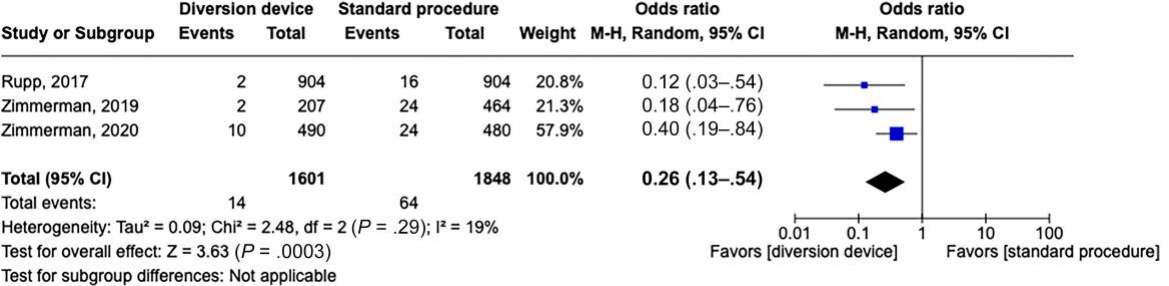
Forest plot of blood culture contamination with a diversion device or a standard procedure of blood collection, in high-quality (Downs and Black ≥18) studies. Odds ratios were determined with the Mantel-Haenszel random-effects method. Abbreviations: CI, confidence interval; M-H, Mantel-Haenszel.

**Figure 3.**
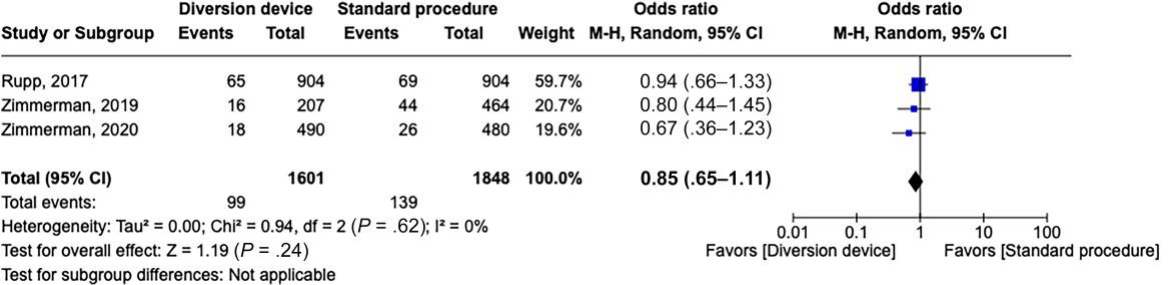
Forest plot of true infection detection with a diversion device or a standard procedure of blood collection. Odds ratios were determined with the Mantel-Haenszel random-effects method. Abbreviations: CI, confidence interval; M-H, Mantel-Haenszel.

When analyzing the 3 studies that were considered of high quality by Downs and Black evaluation (≥18 points) and reported the total number of blood cultures [28, 30, 31], using a stratified analysis, we observed that diversion device adoption was associated with a reduced rate of blood culture contamination (pooled OR, 0.26 [95% CI, .13–.54]) with low heterogeneity results for the studies (*P* = .29; *I*^2^ = 19%) (Figure 2). Conversely, the stratified analysis assessing true infection (3 studies) [28, 30, 31] showed no significant difference (pooled OR, 0.85 [95% CI, .65–1.11]), with homogenous results (*P* = .62; *I*^2^ = 0%) (Figure 3).

The potential for publication bias was assessed using 2 methods. The funnel plot (Supplementary Figure 2) appeared symmetrical and the Egger test did not indicate publication bias among the 9 studies included in the meta-analysis (*P* = .12). When the 3 high-quality studies were assessed by the Egger test, no publication bias was shown either (*P* = .13).

## DISCUSSION

In this systematic literature review and meta-analysis, initial blood specimen diversion devices were associated with a lower frequency of blood culture contamination. Adopting diversion devices may reduce unwanted outcomes, such as unnecessary antibiotic use, prolonged hospital LOS, and mortality. Diversion devices also reduce costs associated with contamination and diagnostic error in healthcare settings.

Diagnostic stewardship involves the responsible use of diagnostic tests to improve patient outcomes while minimizing potential harms to patients and reducing healthcare cost. Diversion devices represent a useful diagnostic stewardship strategy [15, 32]. However, the implementation of diversion devices was not tested in scenarios without standard measures to reduce blood culture contamination (skin and bottle preparation, sterile collection technique, education, and training). Diversion devices should be implemented in conjunction with these strategies to reduce blood culture contamination.

To minimize contamination in blood cultures, a range of methods can be utilized, including proper training of personnel, skin disinfection before venipuncture, avoiding drawing blood from catheters unless necessary, using aseptic techniques, and utilizing prepackaged blood culture kits. According to the Clinical and Laboratory Standards Institute guidelines, the use of paired culture sets is recommended to distinguish between contaminant organisms and true pathogens. For the initial evaluation, it is suggested that four 10-mL bottles (equivalent to 2 sets) should be applied, in order to detect approximately 90%–95% of bacteremias, whereas the bacteremia detection rate of 3 sets is 95%–99% [33]. It is also recommended to regularly monitor contamination rates and report surveillance data to phlebotomists and nurses. However, despite these measures, some microbes may still survive local skin disinfection and accidentally contaminate the blood sample.

Blood culture contamination has many negative impacts on patients. It can lead to misdiagnosis and subsequent inappropriate use of antibiotics, either by prescribing the wrong antibiotic or by extending duration, contributing to the development of antimicrobial resistance. All these events are associated with prolonged hospital stay, which can increase the risk of healthcare-associated infections and complications. Patients may be required to do additional tests, leading to increased healthcare costs and overloading the healthcare system. In this scenario, the adoption of diversion devices could be a valid instrument to reduce untoward outcomes for patients and the healthcare system.

An important finding, and also limitation, of our study is the absence of a consensual definition for blood culture contamination. Two studies by the same research group [30, 31] adopted the same blood culture contamination definition, while another group of 3 studies [20, 28, 29] followed similar definitions for blood culture contamination among them. Clearly there are some tendencies in the inclusion of some species in the definition, such as CoNS, *Micrococcus* spp, *Corynebacterium* spp, α-hemolytic viridans group streptococci, and *Bacillus* spp. However, we believe that a standardization in the definition of blood culture contamination would help the analysis of future studies, making them more methodologically homogeneous to compare. Since the result of a false-positive culture necessarily involves the definition of contamination, the lack of standardization interfered in our comparative analysis and continues to obscure the real extent of blood culture contamination [34]. The College of American Pathologists proposed a definition of blood culture contamination, which is the growth of 1 or more of the following organisms in only 1 blood culture set and only 1 of a series of blood culture sets (eg, 1 of 1, 1 of 2, 1 of 3 sets): CoNS, *Micrococcus* spp, viridans group streptococci, *Propionibacterium acnes*, *Corynebacterium* spp, and *Bacillus* spp [2, 9, 35]. Of the studies that we analyzed, only one-quarter followed this definition [20, 28, 29]. More recently, the Veterans Health Administration microbiology group, with the objective of standardizing the definition of blood culture contamination, published a list of microorganisms whose growth in the culture bottle would be a sign of false-positive result and not true infection [36].

There are only a few diversion devices available on the market. The studies we included analyzed Steripath and Kurin. Despite having similar conclusions of diversion device benefits, Steripath was analyzed by studies of higher quality and clearer methodology than Kurin. An evident advantage of Kurin is the lower diverted blood volume, only 0.15 mL, in comparison to 1.5–2 mL of Steripath. Only 1 of the 3 studies that evaluated Kurin could be included in our meta-analysis since the other 2 did not report the total number of blood cultures, which was essential for the type of analysis we proposed to do. Diversion devices were capable of reducing blood culture contamination and, at the same time, of identifying true infection. In other words, the diversion of the initial blood specimen avoids contamination but does not hinder the detection of a true infection.

Our study has several limitations, mostly due to inherent gaps or constraints in the available primary literature. First, most studies (10) were not randomized [20–24, 26–30]. However, this is the most common study design in the infection prevention literature [37]. Second, most studies were of moderate to low quality and may have overestimated or underestimated the results of blood culture contamination and true infection. Third, there were missing data of important analyzed aspects, such as antisepsis procedure, blood culture contamination definition, and outcomes (antibiotic use, hospital LOS, mortality). Furthermore, the lack of a standard blood culture contamination definition may have affected the homogeneity of our analysis. The quality of the reports of the studies varied, with some studies providing incomplete information about their methods and results. This may have introduced reporting bias into our analysis. However, we present a stratified analysis including high-quality studies showing that diversion devices can prevent blood culture contamination. Fourth, the number of blood cultures was considerably different among the studies, varying from 671 [30] to 41 685 [21], resulting in different weights during some of the stratified analysis. Fifth, some studies inferred outcomes (antibiotic use, hospital LOS, mortality), but not many analyzed them based on their own participants’ data, which precluded our initial intention of analyzing the cost-effectiveness of diversion device adoption. Sixth, the studies included in our analysis were conducted in various settings, including different hospitals, and patient populations, which may have introduced heterogeneity and reduced the generalizability of our findings. Seventh, we only included studies that used a diversion device as the intervention and did not compare diversion devices to other methods of preventing blood culture contamination. This may have limited our ability to draw conclusions about the effectiveness of diversion devices relative to other interventions. Eighth, it was not part of the scope of this systematic literature review to study potential barriers to the adoption of diversion devices, such as staff resistance to change, the need for additional training, and whether the cost of the device systematically adopted is lower than the costs involved in the management of false-positive cases. It also should be noted that despite the limitations of the included studies, the use of a diversion device is not likely to mislead the true diagnosis in blood cultures. However, the studies may not have been designed to specifically address this question, and more research may need to confirm this finding.

Further research is needed to address several important unanswered questions. Specifically, head-to-head studies comparing different diversion devices would provide valuable insights into their relative effectiveness. Studies involving children would help clarify the possibilities and difficulties of applying diversion devices in that population. Additionally, conducting more robust cost-effectiveness studies would help determine the economic impact of implementing diversion devices compared to managing false-positive cases.

In conclusion, the use of diversion devices in healthcare facilities could be a valid instrument to reduce unnecessary antibiotic use and unwanted outcomes to patients and to the healthcare system.

## Supplementary Material

ofad433_Supplementary_Data

## Supplementary Data

Supplementary materials are available at *Open Forum Infectious Diseases* online. Consisting of data provided by the authors to benefit the reader, the posted materials are not copyedited and are the sole responsibility of the authors, so questions or comments should be addressed to the corresponding author.

## Notes

***Acknowledgments*.** We thank Kioko Kusuki de Oliveira, librarian from Instituto Israelita de Ensino e Pesquisa of Hospital Israelita Albert Einstein, for her invaluable assistance in defining our research strategy. This work was partially supported by the National Institutes of Health (grant number 5R25AI147369-03).
