# Supplementary material for: Diagnostic Stewardship: A Systematic Review and Meta-analysis of Blood Collection Diversion Devices Used to Reduce Blood Culture Contamination and Improve the Accuracy of Diagnosis in Clinical Settings
Source: Open Forum Infect Dis. 2023 Aug 11;10(9):ofad433. doi: 10.1093/ofid/ofad433 (PMC10478151; doi:10.1093/ofid/ofad433)
Supplement: ofad433_Supplementary_Data [file ofad433_supplementary_data.docx]

**Supplemental Table 1**: PubMed search strategy

| Group 1 | Group 2 | Group 3 |
| --- | --- | --- |
| venipuncture  OR  phlebotomy  OR  blood specimen collection  OR  blood culture  OR  blood cultures | infection control  OR  diagnostic stewardship  OR  bacteremia  OR  bloodstream infection  OR  (equipment) contamination | device  OR  medical device  OR  initial specimen diversion device  OR  equipment |

((((((((venipuncture[Title/Abstract]) OR (phlebotomy[Title/Abstract])) OR (blood specimen collection[Title/Abstract])) OR (blood culture[Title/Abstract])) OR (blood cultures[Title/Abstract])) AND (((((infection control[Title/Abstract]) OR (diagnostic stewardship[Title/Abstract])) OR (bacteremia[Title/Abstract])) OR (bloodstream infection[Title/Abstract])) OR (contamination[Title/Abstract]))) AND ((((device*[Title/Abstract]) OR (medical device*[Title/Abstract])) OR (initial specimen diversion device[Title/Abstract])) OR (equipment*[Title/Abstract]))) OR (((((((venipuncture[Text Word]) OR (phlebotomy[Text Word])) OR (blood specimen collection[Text Word])) OR (blood culture[Text Word])) OR (blood cultures[Text Word])) AND (((((infection control[Text Word]) OR (diagnostic stewardship[Text Word])) OR (bacteremia[Text Word])) OR (bloodstream infection[Text Word])) OR (contamination[Text Word]))) AND ((((device*[Text Word]) OR (medical device*[Text Word])) OR (initial specimen diversion device[Text Word])) OR (equipment*[Text Word])))) OR ((((infection control[MeSH Terms]) OR (bacteremia[MeSH Terms])) OR (equipment contamination[MeSH Terms])) AND (((blood specimen collection[MeSH Terms]) OR (phlebotomy[MeSH Terms])) OR (blood culture[MeSH Terms])))

**Supplemental Table 2**: Results of stratified analyses

| Study | Analysis | N of studies | Pooled OR (95%CI) | P value | Heterogeneity | |
| --- | --- | --- | --- | --- | --- | --- |
|  |  |  |  |  | I^2^ (%) | P value |
| Blood culture contamination | All studies | 9 | 0.14 (0.09-0.24) | <0.001 | 81 | <0.001 |
|  | Studies published in 2021-2022 | 5 | 0.09 (0.03-0.25) | <0.001 | 91 | <0.001 |
|  | Studies published in 2017-2020 | 4 | 0.21 (0.12-0.34) | <0.001 | 37 | 0.19 |
|  | Duration ≥10 months | 3 | 0.04 (0.01-0.19) | <0.001 | 74 | 0.02 |
|  | Duration <10 months | 6 | 0.21 (0.14-0.31) | <0.001 | 67 | 0.009 |
|  | Non-randomized prospective controlled | 5 | 0.15 (0.10-0.22) | <0.001 | 37 | 0.17 |
|  | Randomized clinical trials | 2 | 0.20 (0.05-0.79) | <0.001 | 85 | 0.01 |
|  | Non-randomized prospective controlled or randomized clinical trials | 7 | 0.16 (0.10-0.23) | <0.001 | 56 | 0.03 |
|  | Quasi-experimental studies | 2 | 0.05 (0.00-12.23) | <0.001 | 97 | <0.001 |
|  | Performed in academic medical centers | 5 | 0.08 (0.02-0.39) | <0.001 | 87 | <0.001 |
|  | Not performed in academic medical centers | 4 | 0.19 (0.12-0.30) | <0.001 | 76 | 0.006 |
|  | Only Steripath testing | 7 | 0.15 (0.08-0.25) | <0.001 | 80 | <0.001 |
|  | Used BacT/Alert | 3 | 0.10 (0.04-0.24) | <0.001 | 76 | 0.02 |
|  | Used BACTEC | 4 | 0.09 (0.01-0.75) | <0.001 | 90 | <0.001 |
|  | Collection by nurses AND phlebotomists | 5 | 0.14 (0.07-0.27) | <0.001 | 82 | <0.001 |
|  | Collection by nurses OR phlebotomists | 4 | 0.14 (0.05-0.43) | <0.001 | 80 | 0.002 |
|  | Collection only by nurses (and nurse assistants) | 2 | 0.13 (0.02-0.98) | <0.001 | 93 | <0.001 |
|  | Collection only by phlebotomists (and resident physicians) | 2 | 0.15 (0.05-0.42) | <0.001 | 0 | 0.72 |
|  | Only patients from emergency departments | 6 | 0.17 (0.10-0.30) | <0.001 | 80 | <0.001 |
|  | More than 1,500 patients in the control group | 4 | 0.09 (0.03-0.26) | <0.001 | 92 | <0.001 |
|  | Less than 1,500 patients in the control group | 5 | 0.18 (0.11-0.30) | <0.001 | 41 | 0.15 |
|  | More than 1,500 patients in the diversion device group | 5 | 0.12 (0.06-0.25) | <0.001 | 89 | <0.001 |
|  | Less than 1,500 patients in the diversion device group | 4 | 0.18 (0.08-0.39) | <0.001 | 56 | 0.08 |
|  | Reported the skin antisepsis technique | 7 | 0.16 (0.10-0.24) | <0.001 | 72 | 0.002 |
|  | Reported the use of chlorhexidine in the skin | 4 | 0.11 (0.05-0.22) | <0.001 | 63 | 0.04 |
|  | Reported the use of alcohol in the skin | 4 | 0.18 (0.08-0.39) | <0.001 | 67 | 0.03 |
|  | Reported the bottle antisepsis technique | 6 | 0.14 (0.10-0.20) | <0.001 | 34 | 0.18 |
|  | Defined blood culture contamination | 9 | 0.14 (0.09-0.24) | <0.001 | 81 | <0.001 |
|  | Made an adjusted analysis for confounders | 4 | 0.13 (0.05-0.35) | <0.001 | 78 | 0.004 |
|  | Studied outcomes | 3 | 0.24 (0.12-0.51) | <0.001 | 76 | 0.01 |
|  | Made financial analysis | 4 | 0.21 (0.14-0.32) | <0.001 | 68 | 0.03 |
|  | High quality studies (D&B ≥18) | 3 | 0.26 (0.13-0.54) | <0.001 | 19 | 0.29 |
| True infection | All studies | 3 | 0.85 (0.65-1.11) | 0.24 | 0 | 0.62 |

**Supplemental Table 3:** Details of Downs and Black (D&B) scale for each study

| Study | Reporting (Max 11 points) | External validity (Max 3 points) | Internal validity – bias (Max 7 points) | Internal validity – confounding (selection bias) (Max 6 points) | Power (Max 1 point) | Total score |
| --- | --- | --- | --- | --- | --- | --- |
| Arenas, 2021 | 4 | 3 | 4 | 2 | 0 | 13 |
| Bell, 2018 | 5 | 2 | 5 | 2 | 0 | 14 |
| Burnie, 2021 | 4 | 2 | 2 | 1 | 0 | 9 |
| Buzard, 2021 | 6 | 2 | 4 | 2 | 1 | 15 |
| Geisler, 2019 | 8 | 2 | 5 | 4 | 0 | 19 |
| Nielsen, 2021 | 4 | 3 | 5 | 3 | 0 | 15 |
| O’Sullivan, 2019 | 4 | 3 | 3 | 1 | 0 | 11 |
| Povroznik, 2022 | 8 | 2 | 3 | 1 | 1 | 15 |
| Rupp, 2017 | 9 | 2 | 5 | 6 | 0 | 22 |
| Tompkins, 2022 | 8 | 2 | 4 | 3 | 0 | 17 |
| Zimmerman, 2019 | 9 | 2 | 4 | 3 | 0 | 18 |
| Zimmerman, 2020 | 9 | 3 | 6 | 4 | 1 | 23 |

**Supplemental Figure 1**: Forest plots

**A: All studies**


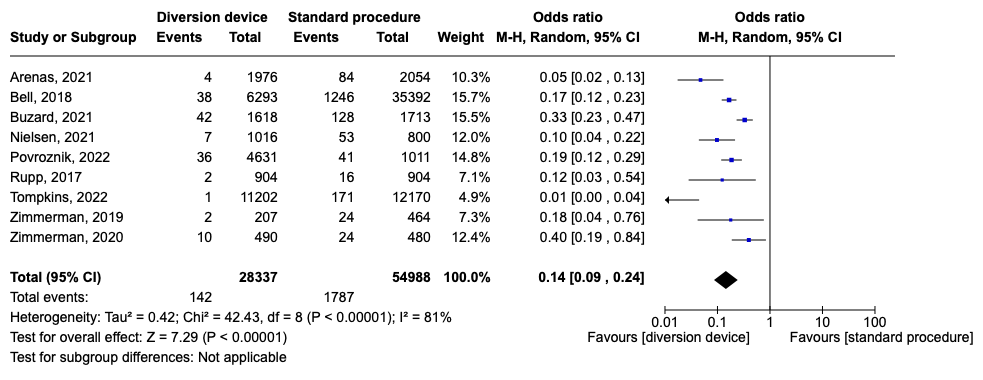


**B: Studies published in 2017-2020**


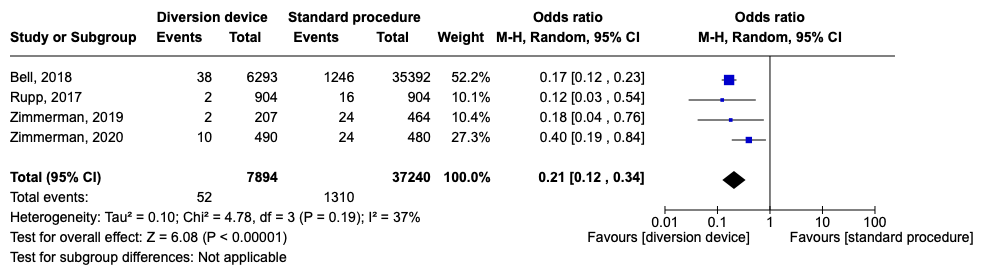


**C: Non-randomized prospective controlled studies**


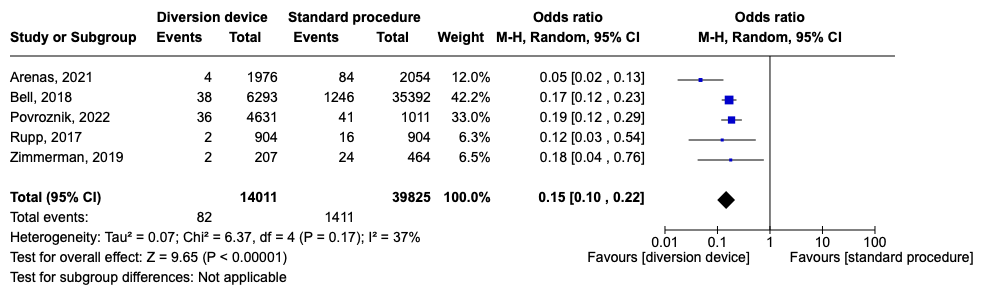


**D: Less than 1,500 participants in the control group**


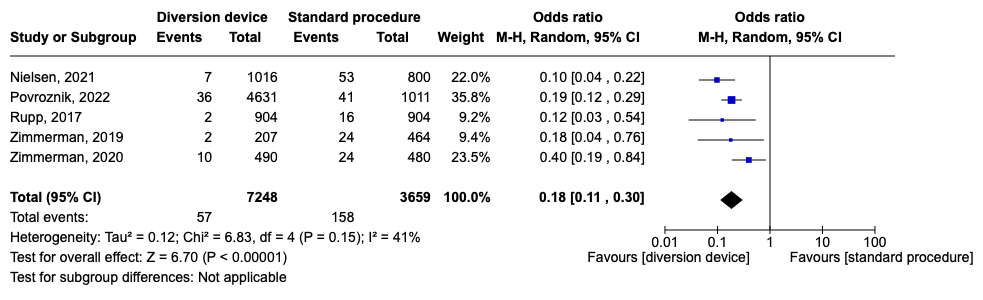


**E: Studies that reported bottle antisepsis technique**


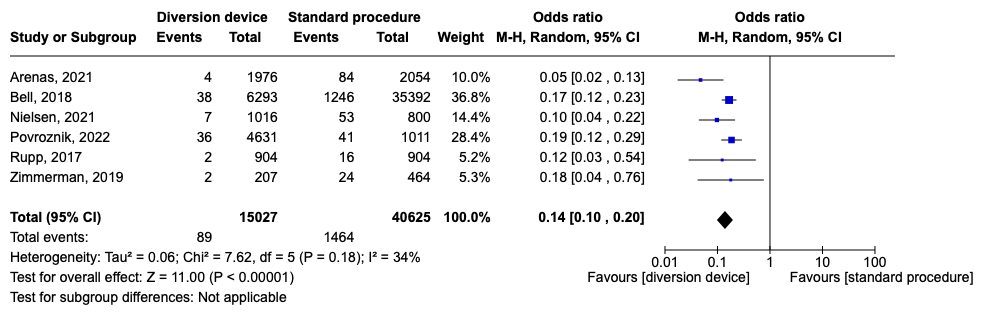


**Supplemental Figure 2**: Funnel plot


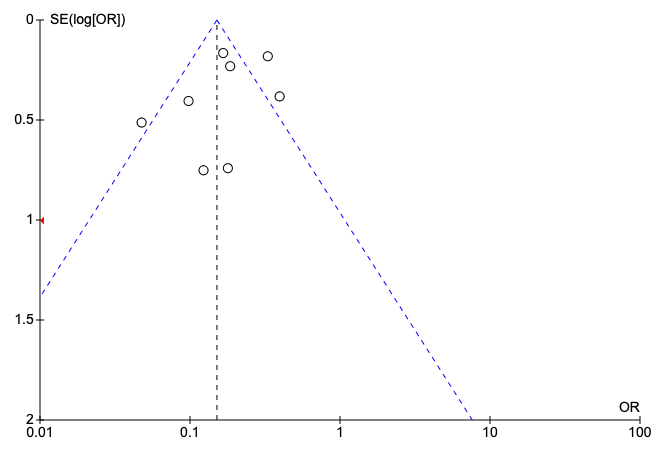


SE: Standard Error, OR: Odds Ratio

**Supplemental Form 1**: Data Abstract Form

1. **Journal:** ____________________________________
2. **First author last name**: __________
3. **Publication year**: __________
4. **Study location (city, state and country)**: __________
5. **Reviewer's initials**: __________

**Part 1**. Basic inclusion/exclusion criteria

1. Did the study evaluate **blood culture contamination**?
   1. Yes
   2. No (If no, exclude it)
2. Did the study evaluate a **device used to minimize the risk of contamination**?
   1. Yes
   2. No (If no, exclude it)
3. If item #2 is "Yes", **which device**?
   1. Steripath® - Initial Specimen Diversion Device (ISDD)®
   2. Other: __________
4. What was the microbial detection system used?
   1. BACT/ALERT
   2. Other: __________
5. How many mL are being diverted by the device? (number) ____ mL
6. Who used the device?
   1. Nurses
   2. Phlebotomists
   3. Other: __________________
7. Did the study evaluate **ways to reduce contamination through changes in traditional blood specimen collection**? (The idea of our study is to evaluate if changes specifically in the **collection** are capable of reducing the blood culture contamination)
   1. Yes (please, describe): ____________________________________
   2. No (If no, exclude it)
8. Duration of the study (in months): __________
9. Do you believe this study should be **excluded**?
   1. Yes
   2. No
10. If "Yes", why? __________________________________________________

**Part 2**. Exposure and outcomes assessment (**check more than one if necessary**)

1. Where did the study take place?
   1. An academic medical center
   2. A community hospital
   3. Other: __________
2. Which type is this **study design**?
   1. Retrospective cohort study
   2. Prospective cohort study
   3. Case-control study
   4. Randomized controlled trial
   5. Other: __________
3. Was the study performed in more than one hospital?
   1. Yes (please write the number of hospitals): _____
   2. No
4. Did the study **compare more than one collection device**?
   1. Yes (number of devices and names): ________________________
   2. Only one collection device (name): __________________________
5. Was the **comparison made with a technique considered to be "traditional"**?
   1. Yes
   2. No (what was the comparison group?): _____________________________ ____________________________________________________________
6. If "Yes", **describe the traditional collection technique:** ____________________________________________________________________________________________________________________________________
7. Do the patients included in the analysis have **a particular feature/belong to a specific group (ethnic, laboral, age, inpatients)**?
   1. Yes
   2. No, they were randomized selected within the general population
8. If "Yes",
   1. Ethnic
   2. Laboral
   3. Etarian
   4. Inpatients
   5. Other (please, specify): _________________________________________
9. How were participants recruited and how were patients selected for the intervention? _______________________________________________________ _________________________________________________________________
10. Describe the **antisepsis technique adopted by the investigators**: ____________ __________________________________________________________________
11. Did the study describe the **blood collection technique using the studied diversion device**?
    1. Yes (please, describe): _________________________________________ ____________________________________________________________
    2. No
12. How did the study define **the concept of blood culture contamination**? _______ __________________________________________________________________
13. Did the study compare **blood culture samples (bottles) from the same patient**?
    1. Yes
    2. No (what instead): _____________________________________________
14. How many mL was collected for one bottle? ____ mL
15. Did the study use as denominator the number of patients or the number of collected blood samples?
    1. The number of patients
    2. The number of collected blood samples
    3. Both
    4. Other denominator (please, explain): ________________
16. Did the study evaluate **the number of blood culture bottles used per patient**?
    1. Yes (how many): ______
    2. No
17. Did the study evaluate **the length of stay in the health care unit and the impact of the diversion device in it**?
    1. Yes (describe the findings): ______________________________________ ____________________________________________________________
    2. No
18. Did the study evaluate **the financial impact of the adoption of the device**?
    1. Yes
    2. No
19. If "Yes", please, summarize the conclusion of the financial analysis: ____________ __________________________________________________________________
20. Population characteristics:

|  | New technique using the device | "Traditional" technique |
| --- | --- | --- |
| Total number |  |  |
| Age (Mean [SD] or Median [IQR]) |  |  |
| % of female |  |  |
| Total number of samples considered to be contaminated |  |  |
| Comorbidities |  |  |
| Length of stay |  |  |
| Mortality |  |  |
| The collection was made from peripheral or central access? |  |  |
| CDI (Clostridium difficile infection) |  |  |
| Venous thromboembolism |  |  |
| Pneumonia |  |  |

**Part 3**. Unadjusted and adjusted associations

1. Raw numbers: Please fill raw data for the following tables if available. Only include measures of effect if they are listed in the article.

Tables: Main association of interest - Positive cultures (total number)

| **Contaminated blood culture** | Positive | Negative |
| --- | --- | --- |
| Control group (traditional technique) |  |  |
| Intervention group (diversion device) |  |  |

| **True infection** | Positive | Negative |
| --- | --- | --- |
| Control group (traditional technique) |  |  |
| Intervention group (diversion device) |  |  |

|  |  |  |
| --- | --- | --- |
| Control group (traditional technique) |  |  |
| Intervention group (diversion device) |  |  |

**Part 4**. Other references

Please look through the references. Are there other references that we should evaluate for the meta-analysis? If yes, please provide first author, journal and year:

________________________________________________________________________________________________________________________________________________________________________________________________________________________

**Part 5**. Quality Assessment Tool

Adapted Downs and Black Tool:

1. Is the hypothesis/aim/objective of the study clearly described?

| Yes | 1 |
| --- | --- |
| No | 0 |

1. Are the main outcomes to be measured clearly described in the Introduction or Methods section?
   1. If the main outcomes are first mentioned in the Results section, the question should be answered no.

| Yes | 1 |
| --- | --- |
| No | 0 |

1. Are the characteristics of the participants included in the study clearly described?
   1. In cohort and cross-sectional studies, inclusion and/or exclusion criteria should be given. In case-control studies, a case-definition and the sources for controls should be given.

| Yes | 1 |
| --- | --- |
| No | 0 |

1. Are the interventions of interest clearly described?
   1. Treatments and placebo (where relevant) that are to be compared should be clearly described.

| Yes | 1 |
| --- | --- |
| No | 0 |

1. Are the distributions of principal confounders in each group of subjects to be compared clearly described?
   1. A list of principal confounders is provided.

| Yes | 2 |
| --- | --- |
| Partially | 1 |
| No | 0 |

1. *Are the main findings of the study clearly described?*
   1. Simple outcome data (including denominators and numerators) should be reported for all major findings so that the reader can check the major analyses and conclusions. (This question does not cover statistical tests which are considered below).

| Yes | 1 |
| --- | --- |
| No | 0 |

1. *Does the study provide estimates of the random variability in the data for the main outcomes?*
   1. In non-normally distributed data the interquartile range of results should be reported. In normally distributed data the standard error, standard deviation or confidence intervals should be reported. If the distribution of the data is not described, it must be assumed that the estimates used were appropriate and the question should be answered yes.

| Yes | 1 |
| --- | --- |
| No | 0 |

1. *Have all important adverse events that may be a consequence of the intervention been reported?*
   1. This should be answered yes if the study demonstrates that there was a comprehensive attempt to measure adverse events. (A list of possible adverse events is provided).

| Yes | 1 |
| --- | --- |
| No | 0 |

1. *Have the characteristics of patients lost to follow-up been described?*
   1. This should be answered yes where there were no losses to follow-up or where losses to follow-up were so small that findings would be unaffected by their inclusion. This should be answered no where a study does not report the number of patients lost to follow-up.

| Yes | 1 |
| --- | --- |
| No | 0 |

1. *Have actual probability values been reported (e.g. 0.035 rather than <0.05) for the main outcomes except where the probability value is less than 0.001?*

| Yes | 1 |
| --- | --- |
| No | 0 |

*External validity:*

*All the following criteria attempt to address the representativeness of the findings of the study and whether they may be generalized to the population from which the study subjects were derived.*

1. *Were the subjects asked to participate in the study representative of the entire population from which they were recruited?*
   1. The study must identify the source population for patients and describe how the patients were selected. Patients would be representative if they comprised the entire source population, an unselected sample of consecutive patients, or a random sample. Random sampling is only feasible where a list of all members of the relevant population exists. Where a study does not report the proportion of the source population from which the patients are derived, the question should be answered as unable to determine.

| Yes | 1 |
| --- | --- |
| No | 0 |
| Unable to determine | 0 |

1. *Were those subjects who were prepared to participate representative of the entire population from which they were recruited?*
   1. The proportion of those asked who agreed should be stated. Validation that the sample was representative would include demonstrating that the distribution of the main confounding factors was the same in the study sample and the source population.

| Yes | 1 |
| --- | --- |
| No | 0 |
| Unable to determine | 0 |

1. *Were the staff, places, and facilities where the patients were treated, representative of the treatment the majority of patients receive?*
   1. For the question to be answered yes the study should demonstrate that the intervention was representative of that in use in the source population. The question should be answered no if, for example, the intervention was undertaken in a specialist center unrepresentative of the hospitals most of the source population would attend.

| Yes | 1 |
| --- | --- |
| No | 0 |
| Unable to determine | 0 |

*Internal validity – bias*

1. *Was an attempt made to blind study subjects to the intervention they have received ?*
   1. For studies where the patients would have no way of knowing which intervention they received, this should be answered yes.

| Yes | 1 |
| --- | --- |
| No | 0 |
| Unable to determine | 0 |

1. *Was an attempt made to blind those measuring the main outcomes of the intervention?*

| Yes | 1 |
| --- | --- |
| No | 0 |
| Unable to determine | 0 |

1. *If any of the results of the study were based on “data dredging”, was this made clear?*
   1. Any analyses that had not been planned at the outset of the study should be clearly indicated. If no retrospective unplanned subgroup analyses were reported, then answer yes.

| Yes | 1 |
| --- | --- |
| No | 0 |
| Unable to determine | 0 |

1. *In trials and cohort studies, do the analyses adjust for different lengths of follow-up of patients, or in case-control studies, is the time period between the intervention and outcome the same for cases and controls ?*
   1. Where follow-up was the same for all study patients the answer should be yes. If different lengths of follow-up were adjusted for by, for example, survival analysis the answer should be yes. Studies where differences in follow-up are ignored should be answered no.

| Yes | 1 |
| --- | --- |
| No | 0 |
| Unable to determine | 0 |

1. *Were the statistical tests used to assess the main outcomes appropriate?*
   1. The statistical techniques used must be appropriate to the data. For example non- parametric methods should be used for small sample sizes. Where little statistical analysis has been undertaken but where there is no evidence of bias, the question should be answered yes. If the distribution of the data (normal or not) is not described it must be assumed that the estimates used were appropriate and the question should be answered yes.

| Yes | 1 |
| --- | --- |
| No | 0 |
| Unable to determine | 0 |

1. *Was compliance with the intervention/s reliable?* Where there was non compliance with the allocated treatment or where there was contamination of one group, the question should be answered no. For studies where the effect of any misclassification was likely to bias any association to the null, the question should be answered yes.

| Yes | 1 |
| --- | --- |
| No | 0 |
| Unable to determine | 0 |

1. *Were the main outcome measures used accurate (valid and reliable)?*
   1. For studies where the outcome measures are clearly described, the question should be answered yes. For studies which refer to other work or that demonstrates the outcome measures are accurate, the ques- tion should be answered as yes.

| Yes | 1 |
| --- | --- |
| No | 0 |
| Unable to determine | 0 |

*Internal validity - confounding (selection bias)*

1. *Were the patients in different intervention groups (trials and cohort studies) or were the cases and controls (case-control studies) recruited from the same population?*
   1. For example, patients for all comparison groups should be selected from the same hospital. The question should be answered, unable to determine for cohort and case- control studies where there is no information concerning the source of patients included in the study.

| Yes | 1 |
| --- | --- |
| No | 0 |
| Unable to determine | 0 |

1. *Were study subjects in different intervention groups (trials and cohort studies) or were the cases and controls (case-control studies) recruited over the same period of time?*
   1. For a study which does not specify the time period over which patients were recruited, the question should be answered as unable to determine.

| Yes | 1 |
| --- | --- |
| No | 0 |
| Unable to determine | 0 |

1. *Were study subjects randomised to intervention groups?* Studies which state that subjects were randomised should be answered yes except where method of randomisation would not ensure random allocation. For example alternate allocation would score no because it is predictable.

| Yes | 1 |
| --- | --- |
| No | 0 |
| Unable to determine | 0 |

1. *Was the randomised intervention assignment concealed from both patients and health care staff until recruitment was complete and irrevocable?*

| Yes | 1 |
| --- | --- |
| No | 0 |
| Unable to determine | 0 |

1. *Was there adequate adjustment for confounding in the analyses from which the main findings were drawn?*
   1. *This question should be answered no for trials if: the main conclusions of the study were based on analyses of treatment rather than intention to treat; the distribution of known confounders in the different treatment groups was not described; or the distribution of known confounders differed between the treatment groups but was not taken into account in the analyses. In non-randomized studies if the effect of the main confounders was not investigated or con- founding was demonstrated but no adjustment was made in the final analyses the question should be answered as no.*

| Yes | 1 |
| --- | --- |
| No | 0 |
| Unable to determine | 0 |

1. *Were losses of patients to follow-up taken into account?*
   1. *If the numbers of patients lost to follow-up are not reported, the question should be answered as unable to determine. If the proportion lost to follow-up was too small to affect the main findings, the question should be answered yes.*

| Yes | 1 |
| --- | --- |
| No | 0 |
| Unable to determine | 0 |

*Power*

1. *Did the study perform calculations to determine sufficient power to detect a clinically important difference?*
   1. *Sample sizes have been calculated to detect a difference of x% and y%.*

| Yes | 1 |
| --- | --- |
| No | 0 |

***Total score: _____***
